# Fitting Accuracy and Constraint Force Measurement of Complete-Arch Implant-Supported Fixed Dental Prostheses Made from Cobalt-Chromium and Zirconia Frameworks Based on the All-on-Four Treatment Concept

**DOI:** 10.3390/ma18184398

**Published:** 2025-09-20

**Authors:** Laura Horsch, Cedric Kirsch, Andreas Zenthöfer, Peter Rammelsberg, Kevin Richter, Stefan Rues

**Affiliations:** Department of Prosthodontics, University of Heidelberg, 69120 Heidelberg, Germany

**Keywords:** biomaterials, dental materials, ceramics, clinical relevance, operative dentistry

## Abstract

The aim of this laboratory study was to evaluate the fitting accuracy of complete-arch implant-supported fixed dental prostheses (ISFDPs) and the occurrence of possible constraint forces after ISFDP fixation using the All-on-four treatment concept. A titanium model was fabricated with support posts for implants in positions 15, 12, 22, and 25. The forces acting on these posts were assessed using strain gauge half bridges. Implants (BEGO Semados^®^ SCX Implantat 4.1 mm × 10 mm, BEGO Implant Systems, Bremen, Germany) were fixated on top of the support posts. Based on conventional impressions and intraoral scans, two 12-unit monolithic ISFDPs made from cobalt–chromium alloy (CoCr) and zirconia (ZrO_2_) were fabricated, jointed with titanium adhesive abutments (PS TiB NH, BEGO), and successively attached to the model. Constraint forces caused by ISFDP fixation were measured for each implant without external force. After testing four ISFDPs with different materials and impression techniques, four new implants were fixated (n = 10 model situations). A standard linear mixed model was used to assess horizontal and vertical constraint forces. The horizontal constraint forces acting on the implants were oriented in the oral direction, indicating that the ISFDPs were too small. The highest constraint forces were measured on implant 22 in the horizontal and vertical directions. Within the limitations of the present laboratory study, the fitting accuracy of complete-arch CoCr and ZrO_2_ ISFDPs based on the All-on-four concept was sufficient for clinical use. Restorations made using conventional impressions had better fitting accuracy and reliability than those made using intraoral scans.

## 1. Introduction

Including a sufficient number of osseointegrated implants in complete-arch prostheses has improved the mechanical performance of restorations and thus the patient health-related quality of life of edentulous patients [1,2]. The All-on-four concept (Nobel Biocare, Goteborg, Sweden) was developed to circumvent the challenges of edentulous jaws and to provide patients with fixed dentures [3]. By the insertion of four implants and the prosthetic treatment with complete-arch implant-supported fixed dental prostheses (ISFDPs), it promises a simple and moderate therapy concept [3,4,5]. Generally, at least four implants are recommended to support a 12-unit maxillary ISFDP [6,7]. Studies have demonstrated promising survival rates for implants under ISFDPs of 98% for 5 years and 95% for 10 years [8,9,10]. Long-term survival has also been demonstrated for complete-arch ISFDPs, usually made from metal alloy frameworks with acrylic prosthesis teeth and veneered gingiva [8,11]. The use of monolithic or partially veneered zirconia (ZrO_2_) is becoming more popular in the All-on-four concept because of its high esthetic value and favorable short-term performances [12,13,14].

However, the mechanical behavior of screw-retained ISFDPs on four implants and their force transmission on the implants has not been well studied. Potential errors or imperfections during the manufacturing process of one-piece long-span ISFDPs could affect the fitting accuracy of ISFDPs. These errors could occur during intraoral scanning (IOS), during the conventional impression (CI) technique, following model cast production, or during computer-aided design/computer-aided manufacturing (CAD/CAM), and can cause clinically relevant inaccuracies that exert constraint forces on the implants when the restorations are screwed in place. The effects of these constraint forces on the implant body and surrounding bone are unknown. The aim of the present laboratory study was to assess the fitting accuracy of 12-unit complete-arch ISFDPs made from cobalt–chromium alloy (CoCr) and ZrO_2_ supported by four implants at positions 15, 12, 22, and 25 in a maxilla model. By measuring constraint forces between implants and restorations that were fixated on adhesive titanium-base abutments, fitting accuracy of the secondary structure could be calculated indirectly. The first study hypothesis was that the fitting accuracies of complete-arch ISFDPs made from CoCr and ZrO_2_ would differ. The second study hypothesis was that the fitting accuracy of ISFDPs would not differ between conventional and digital impression techniques.

## 2. Materials and Methods

### 2.1. Study Design

This was a laboratory study. Experiments were performed using an artificial model of the maxilla, which simulated clinical conditions but did not include biological cells, tissues, or living organisms. This standardized study model of the maxilla was designed with four support posts for implant application (BEGO Semados^®^ SCX Implantat 4.1 mm × 10 mm, BEGO Implant Systems GmbH & Co. KG, Bremen, Germany) at positions 15, 12, 22, and 25, and fabricated from titanium grade 5. Each post was equipped with three strain gauge half bridges. Each post was calibrated with a universal testing device (Z005, Zwick/Roell, Ulm, Germany) and test forces acting in the anterior/posterior, lateral, and vertical direction. This enabled the 3D measurement of constraint forces at each post.

**Figure 1:**
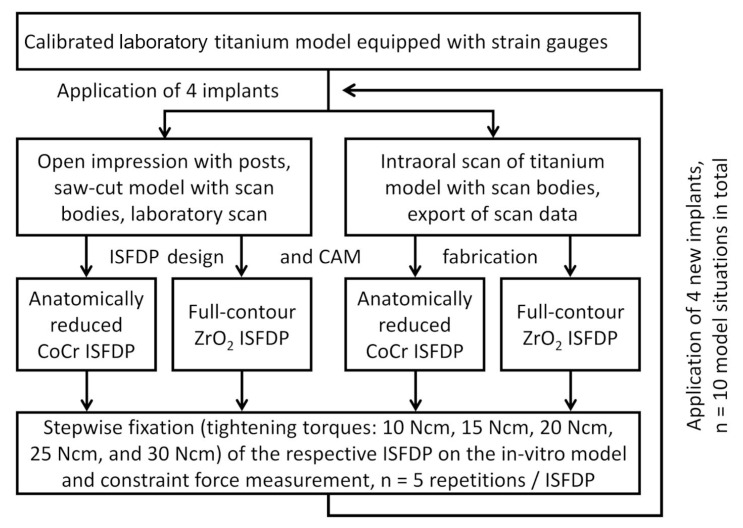
Study flow chart.

Then, CI and IOS (Primescan, Dentsply Sirona, Bensheim, Germany) were performed in n = 10 cases, resulting in 20 scanning cycles. Two 12-unit ISFDPs made from CoCr and ZrO_2_ were CAD/CAM-manufactured for each of the impression techniques. The constraint forces between the model with implants and the restorations screw-retained on the study model were measured to evaluate their fitting accuracy (Figure 1).

### 2.2. Study Model

#### 2.2.1. Design, Manufacturing Process, and Calibration

**Figure 2:**
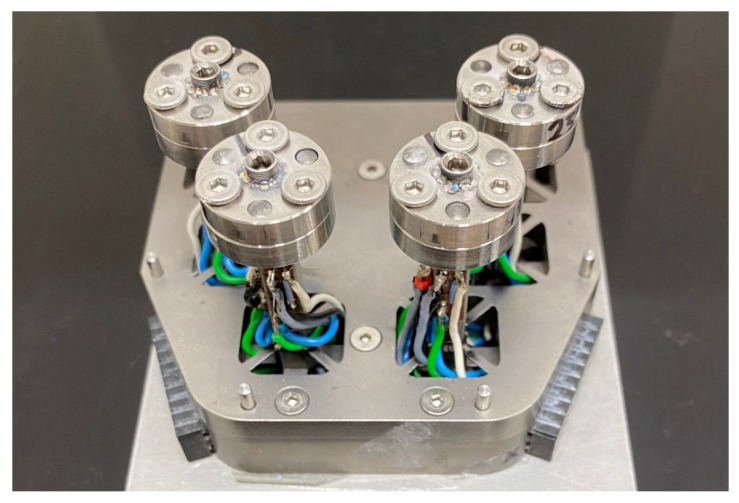
Laboratory model with posts in tooth positions 15, 12, 22, and 25 to accommodate the four implants. Strain gauges were connected via half bridges and positioned on each post to allow measurement of lateral, anterior-posterior, and vertical force components.

No plastic deformations could occur in any component during the tests. Therefore, the support posts and their supporting structures were designed so that an implant fixated at the top of a post had a resilience of 3.7 N/mm in the horizontal direction and 60 mm/N in the vertical direction, which is similar to a natural tooth. Finite element analyses and material parameters of titanium grade 5 (E = 120 GPa, σ_y_ = 830 MPa) have shown that elastic deformations will occur in the support structures at constraint forces < 500 N. This would refer to implant neck deflection thresholds of 1.8 mm in the horizontal direction and 0.3 mm in the vertical direction. The titanium model was equipped with three strain gauge (1-LY13-0.6/120, HBK, Darmstadt, Germany) half bridges at each post (one at the anterior side, one at the lateral side, and one at the basal side) (Figure 2).

Two connected universal amplifying devices (Quantum MX 840B and Quantum MX 440B, HBK, Darmstadt, Germany) provided 12 channels so that signals from all 12 half bridges could be measured simultaneously. Calibration of each direction (x: anterior/posterior, y: lateral, and z: vertical) with test forces between −100 N and +100 N using a 20 N increment and three repetitions/force level processes showed that the mean relative measurement error was <2% and the maximum relative measurement errors was <5%. Forces acting on an implant positioned on top of a post can be converted into implant deflections based on the resilience values, which enabled a more illustrative clinical interpretation of the data.

#### 2.2.2. Implant Fixation and Axis Alignment

Implants were welded to titanium adapter rings (grade V) with vertical orientation. Orientation of the implant was checked based on a 3D scan of the implant with attached scan body (Geomagic Design X, 3DSystems; Morrisville, North Carolina, NC, USA). If the implant axis deviation exceeded the permitted maximum axis deviation of 1.05°, the welding process was repeated until the prerequisites were met. The welded implants were then fixated by fitting pins and screws via their adapter rings onto the posts of the study model.

### 2.3. Impression Techniques

The titanium model was complemented with a printed alveolar ridge and a gingival mask and positioned into a phantom head for the respective impression. CI and IOS were taken for each model situation with four applied implants (n = 10 model situations).

#### 2.3.1. Conventional Impression Using Polyether Material

An individual open tray was designed (Dental Designer,3shape, Copenhagen, Denmark) and 3D-printed (Pro2 UV, Asiga, Alexandria, Australia/Freeprint Tray 2.0, Detax, Ettlingen, Germany). The impression tray rested circularly on the edge of the model so the tray could be positioned identically. Four impression posts (PS OTI, BEGO, Bremen, Germany) were screwed onto the four implants of the study model. The impression tray was conditioned (3M^TM^ Polyether Adhesive, Solventum, MN, USA), and CI was performed in monophasic technology using polyether (Impregum Penta Soft, Solventum, Maplewood, MN, USA). Based on this impression, a precision saw-cut model made from type 4 gypsum (GC Fujirock, Giroform System, Amann Girrbach, Koblach, Austria) was fabricated incorporating laboratory analogs (PS IMPA, BEGO, Bremen, Germany). The saw-cut model was digitized (D2000, 3Shape) with scan bodies (PS CADP, BEGO, Bremen, Germany) placed on top of the analogs.

#### 2.3.2. Digital Impression Using an Intraoral Scanner

**Figure 3:**
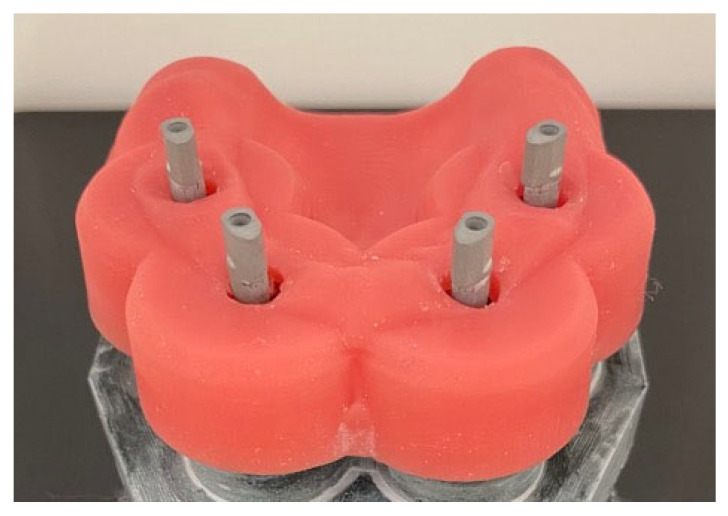
Study model with four implants in positions 15, 12, 22, and 25 and scan bodies (PS CADP, BEGO). Model supplied with printed alveolar ridge and gingival mask.

Prior to the study, 40 IOS impressions were taken of a maxilla model to validate the IOS process quality and to train the examiner. First, the IOS device (Primescan, Dentsply Sirona, Bensheim, Germany) was calibrated, then the implants were given scan bodies (Figure 3). Then, IOS was carried out under the same spatial and lightning conditions without operating light.

### 2.4. Design and Manufacturing of the Frameworks

#### 2.4.1. CAD Design

**Figure 4:**
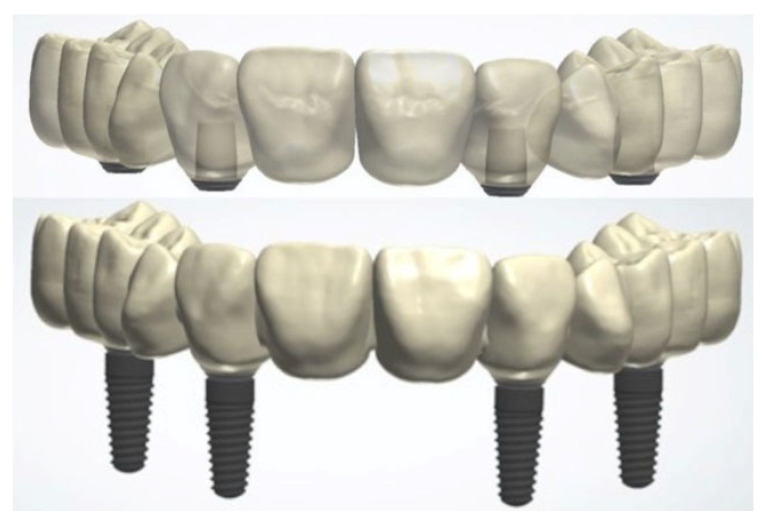
Monolithic ZrO_2_ framework design of complete-arch ISFDPs with adhesive abutments (**top**) and dental implants in positions 15, 12, 22, and 25 (**bottom**).

Based on the CI and IOS data sets for each implant situation, an anatomic 12-unit complete-arch ISFDP was designed for later manufacturing from ZrO_2_ (Dental Designer, 3shape, Kopenhagen, Denmark) (Figure 4).

An anatomically reduced ISFDP was designed for the CoCr frameworks. These were used as templates because the implant positions differed only slightly. For the following model situations, only the locations of the adhesive bases had to be adjusted.

#### 2.4.2. Framework Manufacturing Process

**Figure 5:**
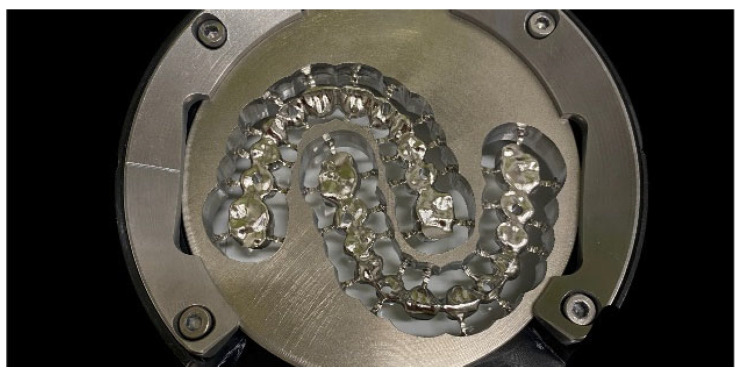
Two fabricated complete-arch ISFDPs after milling from a CoCr blank.

In the next step, ZrO_2_ (IPS e.max ZirCAD LT A2, Ivoclar Vivadent GmbH, Schaan, Liechtenstein) and CoCr frameworks (Wirobond M+, BEGO, Bremen, Germany; Figure 5) were milled for the corresponding model situation (PM7, Ivoclar Vivadent, Schaan, Liechtenstein).

**Figure 6:**
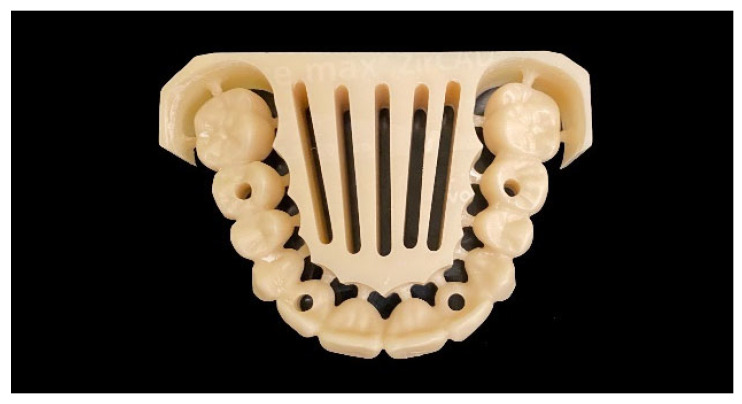
Fabricated complete-arch ISFDPs after milling from a ZrO_2_ blank and sintering with sintering frame still attached.

Cavities for the adhesive bases were milled with a previously adjusted offset parameter (CoCr: −10 µm and ZrO_2_: −12.5 µm) to achieve the best fit between the adhesive base and ISFDP. The parameters were chosen based on preliminary tests of milling, manual fitting, and trial bonding. For the ZrO_2_ ISFDPs, a sintering frame was added during the nesting process. The sintering frame with framework was positioned upright in the furnace (Cercon heat plus, DeguDent GmbH, Hanau, Germany) and was sintered at a final temperature of 1500 °C for 14.5 h (Figure 6).

Bonding bases and frameworks were de-greased with alcohol in preparation for the subsequent bonding process. To enlarge the surface, the adhesive surfaces of abutments and ISFDPs were sandblasted while wearing gloves (2.5 bar, 50 µm aluminum oxide). Surfaces were cleaned with oil-free compressed air and conditioned (Clearfil^TM^ Ceramic Primer Plus, Kuraray Noritake, Tokio, Japan). The adhesive abutments were stuck to the frameworks (Panavia 21, Kuraray Noritake, Tokio, Japan) and stored in an incubator (Function Line B12, Heraeus, Hanau, Germany) for 30 min at 37 °C. For the CI workflow, the adhesive abutments were bonded with ISFDPs using the respective saw-cut model. ISFDPs manufactured using the IOS technique were stuck to the adhesive abutments without an existing model. The internal fit of the frameworks was precisely coordinated in advance so the adhesive bases could also be positioned adequately.

### 2.5. Assessment of Fitting Accuracy by Measuring of Constraint Forces

To see if a final implant position/deflection was reached during ISFDP fixation and to be able to avoid possible damage to the model in case of large inaccuracies (→ preliminary termination of the test in the case of constraint forces exceeding critical thresholds), screws were tightened (ratchet/torque spanner, BEGO Implant Systems) beginning with a tightening torque of 10 Ncm up to a final tightening torque of 30 Ncm, specified by the manufacturer in 5 Ncm increments. With each torque level, screw tightening underwent the same fixation sequence (implant 15 → 12 → 22 → 25). For this reason, no blinding of the impression technique used with the ISFDPs was implemented in the experiments. At the end of each sequence, there was a time period of at least 5 s without any external forces or torques acting on the model so that constraint force could be evaluated. For each model situation, each of the four ISFDP study groups was tested (Figure 1).

To avoid systematic group differences caused by slight model changes due to proceeding measurement of other ISFDPs, a rotating measurement sequence was implemented over ten test repetitions. Each ISFDP was fixated five times on the model as described above to verify data reproducibility. Horizontal constraint forces $F_{h}=\sqrt{F_{x}^{2}+F_{y}^{2}}$ and vertical constraint forces $F_{v}=F_{z}$ were evaluated separately, and average values from five fixation repetitions were used for statistical evaluation.

### 2.6. Statistical Analysis

When planning this study, no reliable data was available for a meaningful sample size calculation. Therefore, this was an explorative investigation. Statistical analysis was performed using SPSS 27 (IBM; New York, NY, USA) and R version 4.4.3 [15]. Bar graphs and box plots were used to visualize the distribution of constraint forces. Linear mixed models were applied to assess the influence of specific factors on both horizontal and vertical constraint forces, including random intercepts to account for repeated measures across scanning cycles. Incorporating the random effects substantially improved model fit: for the model of horizontal forces, the marginal R^2^ was 0.089 and the conditional R^2^ increased to 0.434 (Table 1); for vertical forces, the marginal R^2^ was 0.174 and the conditional R^2^ increased to 0.511 (Table 2). Model diagnostics included inspection of residual plots for normality and variance homogeneity; residuals showed only minor deviations from normality, and no relevant heteroscedasticity was detected. There was no adjustment for multiple testing. *p*-values < 0.05 were considered indicative of statistical significance, but given the exploratory nature of the study, results should be interpreted as hypothesis-generating rather than confirmatory. In addition to statistical significance, the magnitude and potential clinical relevance of effects were considered in the interpretation.

## 3. Results

### 3.1. Development of Constraint Forces on the Implants with Increasing Tightening Torque

At the final tightening torque of M = 30 Ncm, the measured constraint forces showed excellent repeatability with a standard deviation of 0.50 ± 0.35 N. Therefore, average values over the five measurement repetitions were used in all following analyses. To evaluate the development of constraint forces in the horizontal and vertical directions on four implants with increasing screw tightening torque, mean absolute values for each implant position were calculated using the data of all study groups.

**Figure 7:**
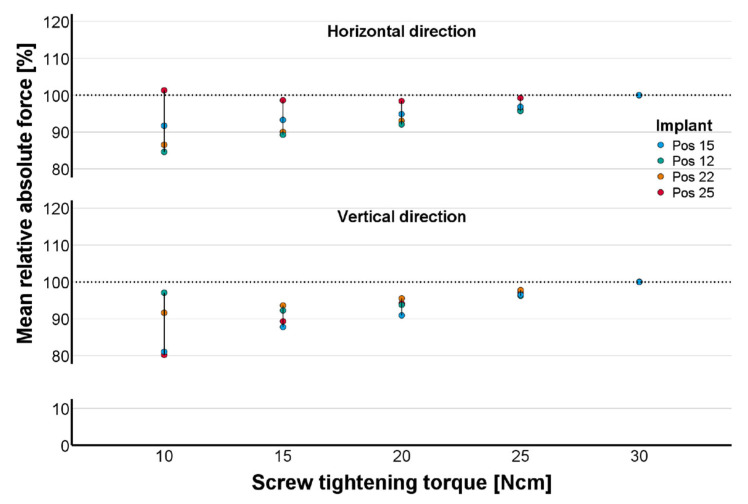
Percentage of absolute constraint forces averaged over data from all study groups. Development of constraint force components in horizontal (top) and vertical (bottom) directions with increasing torque during incremental screw tightening is shown.

The averaged constraint force at the final screw tightening torque of M = 30 Ncm was used as the reference (100%) for all other corresponding values at lower torques. For horizontal and vertical constraint forces, the relative changes recorded between successive torques decreased with increasing tightening torque. Between 25 and 30 Ncm, the relative changes were smaller than 5% for all implant positions (Figure 7). Thus, only minor changes would be expected for even higher screw tightening torques.

### 3.2. Fitting Accuracy of the Restorations Depending on Impression Technique and Framework Material

**Figure 8:**
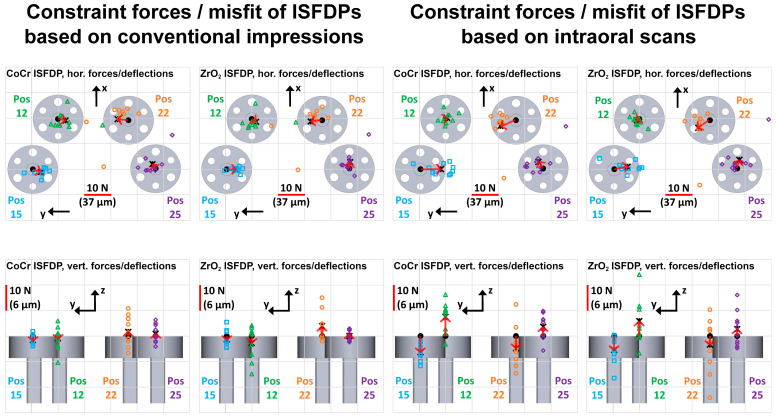
Horizontal (**top**) and vertical (**bottom**) constraint forces of conventionally fabricated ISFDPs on four implant positions (Pos 15, 12, 22, and 25) for all four test groups (CoCr and CI, ZrO_2_ and CI, CoCr and IOS, and ZrO_2_ and IOS). Individual constraint force vectors are displayed as the respective reference position of the implant (black dot) and colored markers. The averaged constraint force vector at each position is also presented (black cross and red arrow). The force vector dimension is shown as a red line (a vector length identical to the grid distance corresponds with a constraint force of 10 N).

The horizontal constraint forces measured for all test groups are displayed in Figure 8. The constraint forces (corresponding post deflections) averaged over each of the ten measurements were plotted as red vectors originating from the respective nominal implant position. These averaged vectors were, in the horizontal direction, mostly oriented along the dental arch or directed towards the central area, indicating that the ISFDPs were too small. In the vertical direction, ISFDP misfits typically resulted in each of the two implants experiencing either tensile or compressive forces. In the CI group, this was rather random, but in the IOS group, the vertical constraint forces followed a pattern—i.e., compressive forces acted on implants in positions 15 and 22, and tensile forces acted on implants in positions 12 and 25.

**Figure 9:**
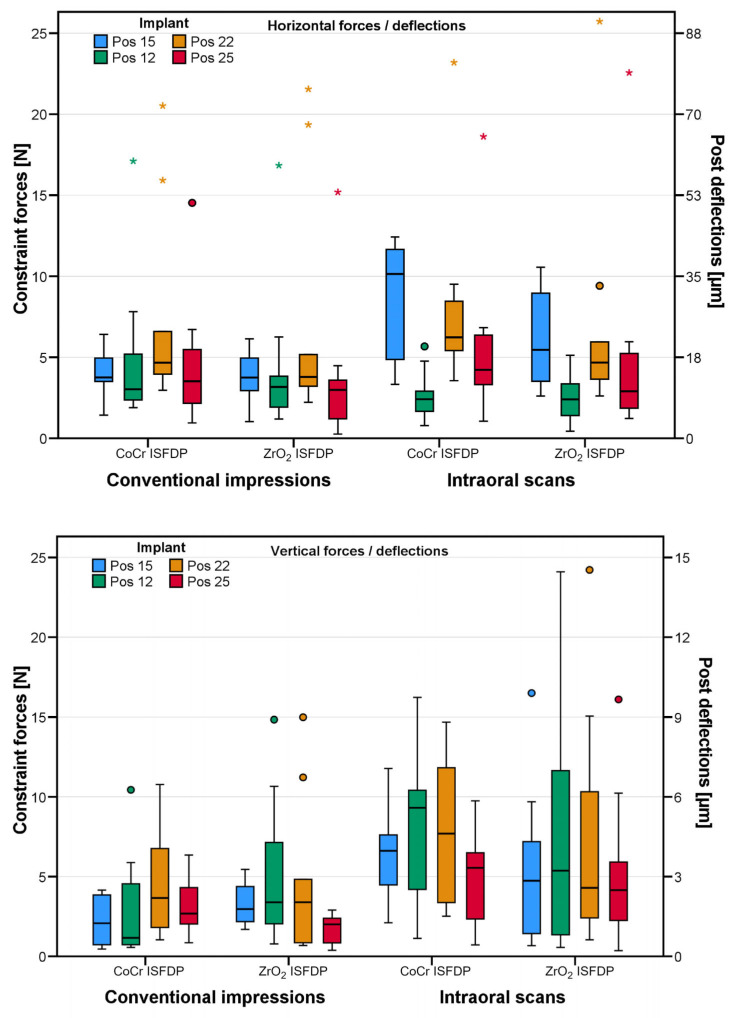
Grouped box-plot diagrams showing horizontal (top) and vertical (bottom) constraint forces/post deflections for both impression methods and implant positions of absolute horizontal and vertical constraint forces. Different implant positions are indicated by different colors. Circles symbolize mild outliers, and asterisks symbolize extreme outliers. ISFDP = implant-supported fixed dental prosthesis, CoCr = cobalt-chrome, and ZrO_2_ = zirconia.

The highest horizontal constraint forces were measured on implant 22 (mean/maximum of 7/25 N corresponding to a deflection of 27/95 µm) (Figure 9). The implant position significantly affected the horizontal constraint force, which was calculated in the linear mixed model. In contrast, the choice of the impression technique and the framework material did not significantly influence the horizontal constraint force (*p* < 0.05; Table 1). In the CI workflow, the absolute constraint forces were below 8 N (corresponding to a deflection of 30 µm) and no predominate bracing direction was observed between two abutments. In the IOS workflow, the highest constraint forces were built up between implants in positions 15 and 22 for both framework materials (Figure 9). An intraclass correlation coefficient of 0.38 indicates the incorporation of scanning cycles as a random effect (Table 1).

The highest vertical constraint forces were measured on implant 22 (mean/maximum of 6/23 N, corresponding to a deflection of 4/15 µm) (Figure 9). The linear mixed model again showed a significant influence of each implant position on constraint forces, whereas the variable framework material did not show a significant effect (*p* = 0.981) (Table 2). In the CI group, the constraint forces did not tend to be in any particular direction at the different positions, with all average constraint forces close to zero. In contrast, in the IOS groups, vertical constraint forces acting on the implants tended to be oriented in the occlusal direction at positions 12 and 25 and in the basal direction at positions 15 and 22 (Figure 8). The use of IOS increased the absolute vertical constraint forces, which may indicate systematic scanning errors with IOS (3.23 N; 95% CI 0.66–5.81 N; *p* = 0.014). An intraclass correlation coefficient of 0.41 indicates the incorporation of scanning cycles as a random effect (Table 2). Therefore, the CI technique might have a better fitting accuracy and greater reliability than IOS. The fit inaccuracies can be greater than the deflections assigned to constraint forces because vertical abutment deflections do not generally compensate for all inaccuracies in the restoration.

## 4. Discussion

The study hypotheses stated that the fitting accuracy of complete-arch ISFDPs differ between restorations made from CoCr and ZrO_2_ but not between restorations made by CI and IOS. Both hypotheses were rejected.

It is difficult to compare the reported fit accuracy of complete-arch ISFDPs between studies because different materials and non-standardized methods have been used. The method of taking measurements by strain gauges on four implant positions presented in this study has been used by others, including the study by Wu et al. [16]. In the present laboratory study, the restorations were fixated starting with a torque value of M = 10 Ncm and then increasing incrementally by 5 Ncm up to maximum of M = 30 Ncm, which was also described in another study [17]. Our findings support the evidence that an absolute passive fit of complete-arch ISFDPs does not exist [18,19,20]. We observed a vertical bracing of the restorations in their final position, independent of the framework. The implant position also influenced this [20]. In the vertical direction, the fitting accuracy and reliability of complete-arch ISFDPs were better in restorations made by CI. This agrees with other studies that recommended using CI rather than complete-arch scans to make long-span restorations [2,21,22].

As zirconia has already been approved for implant-supported short-span restorations and single crowns due to its highly esthetic properties and favorable mechanical behavior [23,24,25], it also became popular in the All-on-four concept [26]. As the manufacturing of ZrO_2_ for complete-arch restorations is more challenging due to its different material-specific properties in contrast to CoCr, the CAD/CAM workflow for ZrO_2_ was experience-based optimized. In the present study, the design included a sintered base and a specific milling strategy under consideration of sintering shrinkage. ISFDPs had to be sintered to full density. In the case of distortions and/or scaling factors not exactly compensating for the real material shrinkage, additional inaccuracies could be associated with this process that can impair the fitting accuracy of the restoration. The results of the present study provide a promising performance of ZrO_2_ for complete-arch ISFDPs, which is in line with previous studies. Authors found similar strain activities and stress behavior of ZrO_2_ compared to titanium and no significant difference in fitting accuracies [18,27]. In contrast to the monolithic design for ISFDPs made from ZrO_2_, restorations made from CoCr must be veneered for esthetic reasons. In the present study, an anatomically reduced design was chosen. For clinical use, these frameworks would have been fully veneered. As porcelain chipping of CoCr ISFDPs is reported as the most frequent major complication in a clinical 5-year observation period; this risk can be reduced by using vestibular veneered ISFDPs made from CoCr [28]. The clinical use of veneered ZrO_2_ is also popular because of esthetic benefits. However, there is an equal risk of chipping, as Larsson et al. reported this complication occurring frequently [26]. As it is recommended for short-span restorations, this complication could be avoided by the reduction in the veneering surface and the reinforced use of monolithic ZrO_2_ [29]. Here, an advantage of ceramic restorations compared to CoCr can be concluded, as they can be designed monolithic or with reduced veneering surface in the anterior teeth area. The body of evidence for long-term data of complete-arch ISFDPs made from ZrO_2_ is limited. Jepsen et al. reported an excellent implant survival and performance of the prosthetic restorations after 5 years of observation [30]. However, because of the scarcity of data and short observation periods, authors recommend, in the latest guideline, removable screw-retained complete-arch ISFDPs and thorough patient education. Protective splints can also be used if periodontal receptors are completely absent, to avoid technical complications [31].

Strength and limitations

Due to the laboratory setup and the model situation in the present study, it was possible to create a situation with ideal- and almost-parallel-positioned implants. The support posts could be planned to simulate a resilience close to the behavior of a natural tooth to exclude any damage or plastic deformations occurring at the implant necks or adhesive bases. By using a 3D-printed alveolar ridge and a rigid gingival mask, attempts were made to simulate the intraoral mucosal situation as close as possible during impression taking. As a difference to the clinical setting, the gingival mask had no influence on the impression and fit of the restorations. Another strength of working with this model was the clarity during impression taking and insertion of the restorations. In contrast, the clinical work can be more challenging due to patient-specific and anatomic aspects. However, the true conditions could only be reflected to a limited extent and the following limitations should be mentioned. Usually, the implant position is often chosen as a compromise between anatomical and prosthetic aspects. Especially in edentulous patients, implant axis divergences are common due to reduced bone supply. Furthermore, blood, saliva and soft tissue may pose difficult conditions during impression taking. The gingiva is often challenging in the clinical impression situation and must be displaced. However, a further limitation of the study is that the movable mucosa was not simulated. The edentulous jaw with movable mucosa is still challenging for IOS because of the lack of landmarks, and this may reduce the fitting accuracy of ISFDPs [32]. As another limitation of the laboratory model, the intraoral biological reactions such as bone remodeling or bone density cannot be reproduced. Due to the presented model situation, the ISFDPs could be (almost) fixated in the final position. In a clinical situation, a lower flexibility of implants must be assumed, such that the frameworks at M = 30 Ncm may not be located in the final position at all implants.

During clinical treatment, successful osseointegration and implant survival cannot be guaranteed, even after sufficient healing time. Especially in posterior areas of atrophic alveolar ridges, tilted implants are used under complete-arch ISFDPs to reduce compressive stresses and increase the tensile stresses of peri-implant bone [33,34]. Therefore, posterior tilted implants and abutments could be tested on the laboratory model. These findings could help to adjust the framework designs, and complete-arch ISFDPs on tilted and non-tilted implants could be investigated in the clinical setting.

## 5. Conclusions

Within the limitations of this laboratory study, we conclude the following:

- Fitting accuracies of complete-arch ISFDPs made from CoCr and ZrO_2_ do not differ significantly.
- Implant position significantly affects absolute constraint forces in the horizontal and vertical direction and therefore affects fitting.
- Restorations made with the CI technique have better fitting accuracy and reliability than restorations made with IOS.
- Further studies are needed to confirm the presented results.

## Figures and Tables

**Table 1:** Linear mixed model for the absolute horizontal constraint forces according to impression technique (CI, IOS), ISFDP material (CoCr, ZrO_2_), and implant position (15, 12, 22, 25). 95% CI = 95% confidence interval; *p*-values are not adjusted. σ^2^ = residual variance, τ_00 scanning cycles_ = random effects variance, and ICC = intraclass correlation coefficient.

Absolute Horizontal Constraint Forces					
Predictors			Estimates	95% CI	*p*
ISFDP material	Reference: CoCr	ZrO_2_-ref	−0.77	−1.91 to 0.37	0.184
Impression technique	Reference: CI	IOS-ref	0.74	−2.02 to 3.50	0.599
Implant position	Reference: Pos 15	Intercept	4.75	2.49–7.01	<0.001
		Pos 12-ref	2.57	0.95–4.18	0.002
		Pos 22-ref	−1.10	−2.71 to 0.51	0.178
		Pos 25-ref	0.83	−0.79 to 2.44	0.313
	Reference: Pos 12	Intercept	7.32	5.06–9.58	<0.001
		Pos 22-ref	−3.67	−5.28–2.06	<0.001
		Pos 25-ref	−1.74	−3.35 to −0.13	0.034
	Reference: Pos 22	Intercept	3.65	1.39–5.91	0.002
		Pos 25-ref	1.93	0.32–3.54	0.019
		σ^2^	13.30		
		τ_00_	8.10		
		ICC	0.38		
		Marginal R^2^ Conditional R^2^	0.089 0.434		

**Table 2:** Linear mixed model for the absolute vertical constraint forces according to impression technique (CI and IOS), material (CoCr and ZrO_2_), and implant position (15, 12, 22, and 25). 95% CI = 95% confidence interval, *p*-values are not adjusted. σ^2^ = residual variance, τ_00 scanning cycles_ = random effects variance, and ICC = intraclass correlation coefficient.

Absolute Horizontal Constraint Forces					
Predictors			Estimates	95% CI	*p*
ISFDP material	Reference: CoCr	ZrO_2_-ref	−0.01	−1.02 to 1.00	0.981
Impression technique	Reference: CI	IOS-ref	3.23	0.66–5.81	0.014
Implant position	Reference: Pos 15	Intercept	2.17	0.09–4.25	0.041
		Pos12-ref	2.46	1.03–3.89	0.001
		Pos 22-ref	2.17	0.74–3.60	0.003
		Pos 25-ref	0.54	−0.89–1.97	0.454
	Reference: Pos 12	Intercept	4.63	2.55–6.71	<0.001
		Pos 22-ref	−0.29	−1.72 to 1.14	0.686
		Pos 25-ref	−1.92	−3.35–−0.49	0.009
	Reference: Pos 22	Intercept	4.34	2.26–6.42	<0.001
		Pos 25-ref	−1.63	−3.05–−0.20	0.026
		σ^2^	10.46		
		τ_00_	7.19		
		ICC	0.41		
		Marginal R^2^ Conditional R^2^	0.174 0.511		

## Data Availability

The data presented in this study are available on request from the corresponding author due to the lack of suitable repositories.

## Abbreviations

The following abbreviations are used in this manuscript.

ISFDPs	implant-supported fixed dental prostheses
CI	conventional impression
IOS	intraoral scan
CoCr	cobalt-chronium alloy
ZrO_2_	zirconia
CAD/CAM	computer-aided design/computer-aided manufacturing
